# The *pcz1* Gene, which Encodes a Zn(II)_2_Cys_6_ Protein, Is Involved in the Control of Growth, Conidiation, and Conidial Germination in the Filamentous Fungus *Penicillium roqueforti*

**DOI:** 10.1371/journal.pone.0120740

**Published:** 2015-03-26

**Authors:** Carlos Gil-Durán, Juan F. Rojas-Aedo, Exequiel Medina, Inmaculada Vaca, Ramón O. García-Rico, Sebastián Villagrán, Gloria Levicán, Renato Chávez

**Affiliations:** 1 Departamento de Biología, Facultad de Química y Biología, Universidad de Santiago de Chile, Santiago, Chile; 2 Departmento de Química, Facultad de Ciencias, Universidad de Chile, Santiago, Chile; 3 GIMBIO Group, Department of Microbiology, Faculty of Basic Sciences, Universidad de Pamplona, Pamplona, Colombia; Georg-August-University of Göttingen Institute of Microbiology & Genetics, GERMANY

## Abstract

Proteins containing Zn(II)_2_Cys_6_ domains are exclusively found in fungi and yeasts. Genes encoding this class of proteins are broadly distributed in fungi, but few of them have been functionally characterized. In this work, we have characterized a gene from the filamentous fungus *Penicillium roqueforti* that encodes a Zn(II)_2_Cys_6_ protein, whose function to date remains unknown. We have named this gene *pcz1*. We showed that the expression of *pcz1* is negatively regulated in a *P*. *roqueforti* strain containing a dominant active Gαi protein, suggesting that *pcz1* encodes a downstream effector that is negatively controlled by Gαi. More interestingly, the silencing of *pcz1* in *P*. *roqueforti* using RNAi-silencing technology resulted in decreased apical growth, the promotion of conidial germination (even in the absence of a carbon source), and the strong repression of conidiation, concomitant with the downregulation of the genes of the central conidiation pathway *brlA*, *abaA* and *wetA*. A model for the participation of *pcz1* in these physiological processes in *P*. *roqueforti* is proposed.

## Introduction

Zinc-binding proteins are one of the largest families of transcription regulators in eukaryotes, displaying structural and functional diversity. According to their zinc finger binding motifs, zinc-binding proteins are classified into three main groups: Cys_2_His_2_, Cys_4_, and Zn(II)_2_Cys_6_ [1]. Interestingly, Zn(II)_2_Cys_6_ proteins (hereafter C6) have been found only in fungi and yeasts, and they seem to be absent from bacteria, plants, and animals [2].

Although the C6 genes are abundant in fungal genomes, mainly from the phylum *Ascomycota*, comparatively few of them (approximately 30–40 genes) have been functionally characterized [2]. From these studies, mainly performed in *Saccharomyces cerevisiae* and model species from the genus *Aspergillus*, it has been deduced that C6 proteins are mainly involved in the regulation of the utilization of carbon and nitrogen compounds, the regulation of secondary metabolism, and the regulation of sexual and/or asexual development [2]. Known examples of C6 proteins participating in these processes are the *S*. *cerevisiae* Gal4 transcriptional activator, which is involved in galactose catabolism [3], the AflR protein controlling the expression of several genes involved in the production of the secondary metabolites aflatoxin and sterigmatocystin in *Aspergilli* [4], and the SfgA protein, a repressor that interacts with FluG, a protein necessary for the activation of conidiation in *A*. *nidulans* [5].

In contrast with yeast and *Aspergillus*, very few C6 proteins have been studied in *Penicillium* species. In the dimorphic fungus *Penicillium marneffei* (currently known as *Talaromyces marneffei*), disruption of the *facB* gene downregulates the expression of the gene encoding for isocitrate lyase, a key enzyme in the glyoxylate cycle [6]. In *P*. *citrinum*, disruption of the mlcR gene demonstrated its involvement in the biosynthesis of the secondary metabolite compactin [7]. Another C6 gene from *P*. *citrinum* (ariB) was disrupted, but its absence was not associated with any phenotypic effect [7]. To our knowledge, no other studies of functional characterization of any C6 protein in *Penicillium* species have been performed.

*Penicillium roqueforti* is a filamentous fungus that is important to the food industry because it is used in the ripening of blue-veined cheeses. However, despite its biotechnological importance, not much progress has been made in understanding the physiology of this fungus. Previously, we have made efforts to gain insight into the biology of *P*. *roqueforti* by studying the effect of a dominant active α subunit in subgroup I (Gαi) of a heterotrimeric G protein [8–10]. Heterotrimeric G proteins remain inactive when their three subunits (Gα and the βγ dimer) are together. In this inactive state, Gα keeps GDP (guanosine 5`- diphosphate) bound, but when a suitable stimulus is sensed, Gα exchanges GDP for GTP (guanosine 5`- triphosphate), resulting in its separation from the βγ dimer. Then, both the Gα and the βγ dimer become active, interacting with downstream effectors. Normally, activation ends when the GTPase activity in Gα hydrolyzes GTP to GDP, causing Gα and the βγ dimer to reassociate [11]. However, in fungi, the replacement of glycine with arginine at the appropriate position produces constitutive activation of Gα, thereby resulting in persistent and dominant active Gα signaling [12].

Transformants of *P*. *roqueforti* containing the dominant active Gαi protein described above showed several phenotypic alterations compared with the wild-type strain: a drastic reduction in conidiation, the ability to germinate their spores in the absence of a carbon source, and delayed apical growth [8–10]. Additionally, these transformants have increased concentrations of cAMP [9]. However, in addition to increases in this metabolite, other putative downstream effectors of Gαi have not been functionally described in *P*. *roqueforti* to date. We hypothesized that the effects of the Gαi protein on the phenotype of the fungus could be due (at least in part) to transcriptional changes in effector genes downstream of the Gαi protein. In this paper, we have characterized a novel gene encoding a putative C6 protein of unknown function, which we have named *pcz1*. We confirmed that the expression of *pcz1* is downregulated in strains of *P*. *roqueforti* containing the dominant active Gαi subunit. More interestingly, a functional analysis of *pcz1* suggests that it may be a positive regulator of conidiation, which is concomitant with the downregulation of the genes of the central conidiation pathway *brlA*, *abaA* and *wetA*. In addition, *pcz1* may be a positive regulator of apical growth, but it may be a repressor of conidial germination.

## Materials and Methods

### Fungal strains and culture media

The wild type strain *P*. *roqueforti* CECT 2905 was kindly provided by Dr. Juan F. Martín (Inbiotec, León, Spain). *P*. *roqueforti* transformants pga5 and pga7, derived from strain CECT 2905 and containing the dominant active Gαi subunit, have been previously described [8].

The media used in this work were potato dextrose agar (PDA; Merck, Darmstadt, Germany), Czapek minimal medium, Czapek yeast extract agar (CYA), yeast extract sucrose (YES), Power medium agar [13] and CM (glucose 5 g/l, yeast extract 5 g/l and malt extract 5 g/l).

### Construction of plasmid pC6-RNAi for *pcz1* silencing

Plasmid pJL43-RNAi [14] was used to generate the *pcz1* knockdown construct. This plasmid contains two promoters in opposite directions, which generate double-stranded RNA molecules (dsRNAs) from a small DNA fragment inserted in an *Nco*I site between these two promoters [14]. These dsRNAs are recognized and processed by the fungal RNA-silencing machinery, resulting in the specific degradation of target mRNAs [14]. Plasmid pJL43-RNAi has already been successfully used for the silencing of genes in *P*. *roqueforti* [15].

Plasmid pJL43-RNAi was digested with *Nco*I. In parallel, a 446-bp fragment of the *pcz1* gene was amplified with primers RNAiC6FW (5`- CAGAAGAGGTCCATGGTC-3`) and RNAiC6RV (5`- AGACTCCCATGGCCAACCGTTGTCGCTG-3`) and was also digested with *Nco*I. The digested fragment (432 bp) was ligated into pJL43-RNAi, thus giving rise to plasmid pC6-RNAi, which was used to transform *P*. *roqueforti* CECT 2905.

### Transformation of *P*. *roqueforti*

*P*. *roqueforti* transformants M9 and M11, with a silenced *pcz1* gene, were obtained by introducing plasmid pC6-RNAi into strain CECT 2905 by protoplast transformation. Protoplast obtainment, transformation, and selection of transformants on Czapek-sorbitol medium containing phleomycin were carried out as described by Chávez et al. [16] except that 10 μg/mL phleomycin was used to select transformants. After transformation, conidia from selected colonies were subsequently transferred three times onto a suitable medium to stabilize the genotype and obtain homokaryotic strains. The same procedure was used to obtain a *P*. *roqueforti* transformant containing empty plasmid pJL43-RNAi, which was used as a control in the experiments.

### DNA and RNA extractions, RT-PCR experiments and suppression subtractive hybridization (SSH) experiments

For DNA isolation, spores from *P*. *roqueforti* strains were inoculated into CM medium at 28°C for 24 hours at 200 r.p.m. in an orbital shaker. Mycelia were harvested by filtration and washed with 0.9% NaCl. DNA from each mycelium was isolated according to Bainbridge et al. [17].

For RNA isolation, mycelia growing under suitable conditions (see the respective Figure legend for details) were harvested and washed as above. Then, they were frozen in liquid nitrogen and ground in a mortar. The total RNA was extracted using the RNeasy Plant Minikit (Qiagen, Germany) according to the manufacturer’s instructions, and treated with RNase-free DNase I (Roche, Germany). Total RNA was quantified in a MultiSkan GO quantification system using a μDrop plate (Thermo Scientific, Germany) according to the manufacturer’s instructions. One μg of total RNA was used to synthesize cDNA using RevertAid Reverse Transcriptase (Thermo Scientific, Germany) according to the manufacturer’s instructions.

RT-PCR experiments were performed essentially as described by Ravanal et al. [18]. PCR conditions were the same as described by Diaz et al. [19]. Primers used for preliminary RT-PCR analyses of *pcz1* were RNAiC6FW and RNAiC6RV (described above). As control, amplification of β-tubulin cDNA was performed with primers Bt2A (5`- GGTAACCAAATCGGTGCTGCTTTC-3`) and Bt2B (5`- ACCCTCAGTGTAGTGACCCTTGGC-3`). Transcription levels were estimated by densitometry analysis using the “myImageAnalysis Software” program (Thermo Scientific, Germany).

For SSH experiments, spores from *P*. *roqueforti* CECT 2905 (wild-type strain) and *P*. *roqueforti* pga7 (containing the dominant active Gαi subunit) were inoculated into YES medium at 28°C for 10 days at 200 r.p.m in an orbital shaker. Mycelia were harvested and washed three times with PBS (137 mM NaCl, 2.7 mM KCl, 10 mM Na_2_HPO_4_, 1.8 mM KH_2_PO_4_, pH 7.4). Total RNA from each mycelium was isolated as above. PolyA+-RNA was purified from total RNA using the “Absolutely mRNA Purification” kit (Stratagene, USA), according to the manufacturer’s specifications and used for SSH. SSH experiments were performed using the “PCR-Select cDNA subtraction” kit (Clontech, USA) and following the manufacturer’s instructions exactly. cDNA from the wild-type strain was utilized as “tester” sample and cDNA from *P*. *roqueforti* pga7 was utilized as “driver” sample. The selectively amplified cDNA molecules were ligated into pGEM-T Easy and transformed into high efficiency competent *E*. *coli* cells. Analysis and selection of bacterial colonies containing plasmids suitable for sequencing were performed by PCR and dot blot assays exactly as described by Klagges et al. [20]. Selected plasmids were sequenced (both strands) by Macrogen (Seoul, Korea).

### Quantitative reverse-transcriptase polymerase chain reaction (qRT-PCR) analysis

Gene expression was analyzed by qRT-PCR. Total RNA quantification and cDNA synthesis were performed as above. For details of specific primers used in each qRT-PCR experiment, see S1 Table. All primer sets exhibited suitable efficiency as required for the comparative Ct (ΔΔCt) method (S2 Table). Reactions were performed in 20 μl reaction volumes. Each reaction contained 10 μl of KAPA SYBR Fast qRT-PCR Master Mix 2x (Kapa Biosystems, USA), 0.4 μl of each primer (at a concentration of 10 μM each), 0.4 μl de 50x ROX High/Low, 6.8 μl of water, and 2 μl of cDNA. Quantification was carried out with a StepOne Real-Time PCR System (Applied Biosystems, USA) using the following conditions: 30 s at 95°C and 40 cycles of 3 s at 95°C and 30 s at 60°C. Appropriate negative controls were included. For each gene expression analysis, three replicates were performed. The data were analyzed according to the comparative Ct (ΔΔCt) method and were normalized to β-tubulin gene expression in each sample.

### Measurement of fungal apical extension rates

Apical extension rates were determined as described by Ivey et al. [21]. Briefly, the diameter of the colony was measured daily in different culture media (see the respective Figure legend for details). In each case, the apical extension rate was obtained as a linear regression of colony diameter over time.

### Measurement of conidial production kinetics

The production of conidia was measured according to García-Rico et al. [8]. Briefly, 100 μl of a conidial suspension (5 × 10^5^ conidia/ml) was seeded in Petri dishes containing a suitable culture medium (see the respective Figure legend for details). Dishes were incubated at 28°C for 1, 3 or 5 days, and the conidia produced were collected by adding NT solution (0.9% NaCl, 0.05% Triton) and scratching the surface of the plate with an inverted Pasteur pipette. This procedure was repeated one time. Conidia obtained were counted in a Neubauer chamber. Values are expressed as conidia/mm^2^ of surface.

### Analysis of conidial germination

With slight modifications, the measurement of conidial germination was performed as previously described [10]. For each strain, three replicate flasks containing 2 x 10^5^ conidia/ml of a suitable liquid media were incubated at 28°C for a variable amount of time (see the respective Figure legend for details about the medium and time). At regular intervals, samples of 10 μl were taken, observed in the microscope, and the number of germinated and non-germinated conidia was counted in 10 randomly-chosen fields (around 230 conidia were counted in each experiment). This procedure was repeated, in duplicate, for each flask (technical replicate). Conidia were considered germinated when the length of their germ tubes were the same size or longer than the diameter of the conidia. Data were plotted as the percentage of germination versus time.

## Results

### The *pcz1* gene is downregulated in *P*. *roqueforti* carrying the dominant Gα subunit

As was stated in Introduction, the effects of the Gαi protein on the phenotype of *P*. *roqueforti* could be due to transcriptional changes in effector genes downstream of the Gαi protein. Therefore, to begin unveiling genes from *P*. *roqueforti* that are regulated by Gαi, we performed SSH experiments (see Materials and methods for details). Among the sequences obtained from these experiments, a cDNA sequence that was putatively differentially expressed in wild-type *P*. *roqueforti* compared with transformants carrying the dominant Gαi protein (S1 Fig.) drew our attention. This sequence matched an unnamed ORF from *P*. *roqueforti* (Proq08g087160; [22]) that encodes a putative C6 protein (see below) of unknown function. A scan of the *P*. *roqueforti* genome indicates that only one copy of this ORF is present (data not shown). Because C6 proteins are exclusive to fungi and have been poorly studied, we decided to characterize this gene, for which the name *pcz1* (for *P* *enicillium* C6 zinc domain protein 1) is proposed.

**Fig 1:**
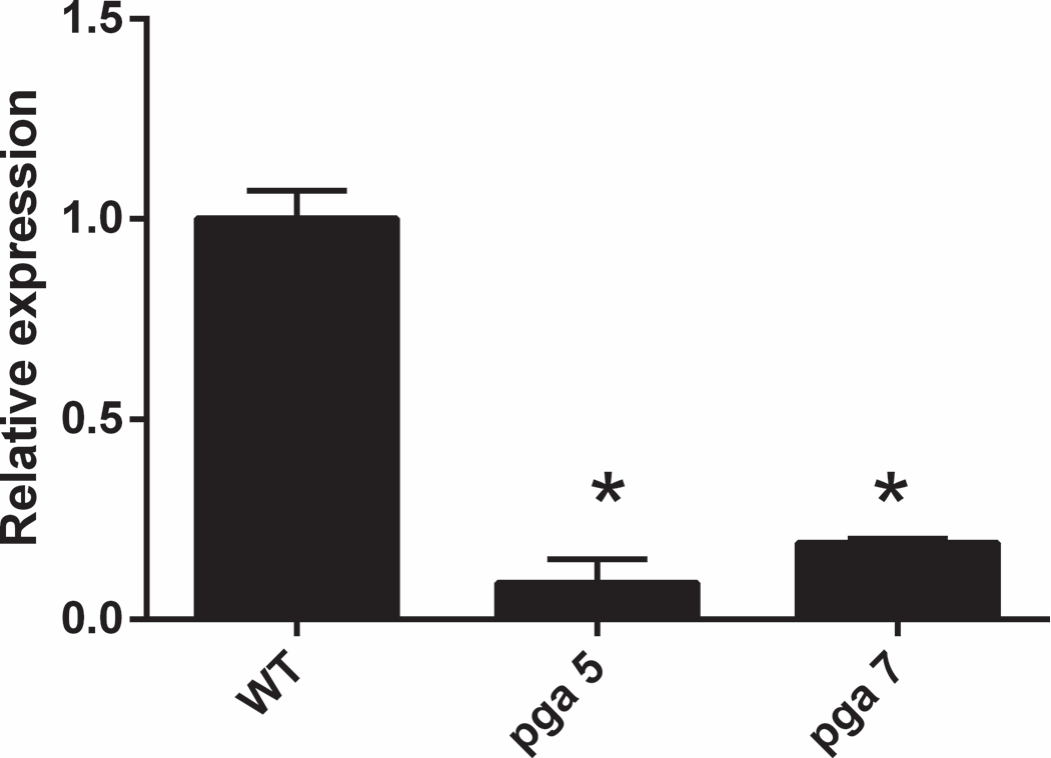
qRT-PCR analysis of the expression of *pcz1* in *P*. *roqueforti* CECT 2905 (WT) and two strains (pga7 and pga5) carrying the dominant Gαi allele. The RNA used was extracted as described in Materials and Methods from strains grown 5 days in CM medium. See Materials and Methods for other details. Error bars represent the standard deviation of three replicates in three different experiments. The differences were considered statistically significant at *P* < 0.05 (*) by using the Student’s *t*-test.

First, we confirmed that *pcz1* was differentially expressed in *P*. *roqueforti* CECT 2905 with respect to the transformants pga5 and pga7, carrying the dominant Gαi protein. Fig. 1 shows the results of qRT-PCR experiments for this gene in these strains. As can be observed, and compared with the wild-type strain, *pcz1* is downregulated in transformants carrying the dominant Gαi subunit. The transformants pga5 and pga7 showed approximately 12.6- and 5.2- fold decrease in *pcz1* transcripts compared with *P*. *roqueforti* CECT 2905. These results suggest that the active Gαi subunit exerts a repressor effect on the expression of *pcz1*.

### Analysis of Pcz1 deduced protein

*pcz1* has previously been annotated and encodes a 790 amino acid protein [22]. BlastP analysis of the deduced Pcz1 protein reveals the presence of orthologs with high levels of similarity in a wide range of species of filamentous fungi from the phylum *Ascomycota*, particularly the classes Eurotiomycetes, Sordariomycetes, Leotiomycetes and Dothideomycetes (data not shown).

The analysis of Pcz1 reveals that the putative Zn_2_(II)Cys_6_ DNA binding domain is at amino acid positions 393–430. A multiple alignment performed with Pcz1 from *P*. *roqueforti* and orthologues from 81 other fungal species revealed that they share the highly conserved motif R-K-L-R-A-C-L-R-C-K-F-L-K-K-T-C-D-[KT]-G-[DE]-P-C-[ATGN]-G-C-[QKR]-P-S-H-A-R-L-W-[QM]-V-P-C-T, where most of amino acids, including the six key cysteines (underlined), are fully conserved (S2 Fig.).

### RNA-mediated gene-silencing of *pcz1*

**Fig 2:**
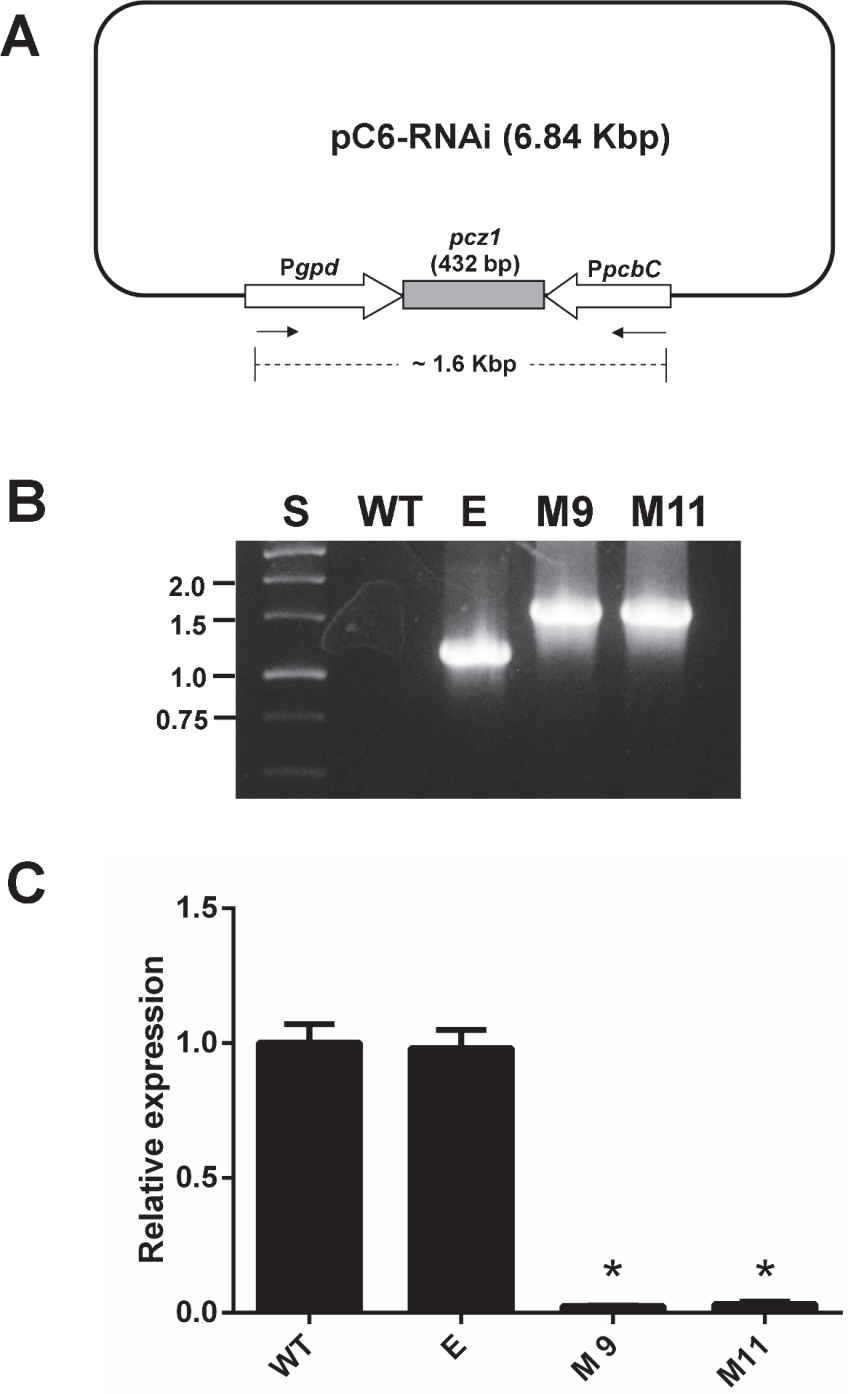
Gene silencing of *pcz1* in *P*. *roqueforti*. (A) Schematic representation of the full silencing cassette in plasmid pC6-RNAi. Pgpd and PpcbC represent the convergent promoters from the *gpd* gene from *A*. *nidulans* and the *pcbC* gene from *P*. *chrysogenum* [14]. The small black arrows represent primers ConfRNAiFW (5`- GCATGCCATTAACCTAGG-3`) and ConfRNAiRV (5`-ACGGTGGCTGAAGATTC-3`), which were used to confirm the integration of the full silencing cassette (see panel B). The expected size of the amplicon containing the full silencing cassette is shown. For simplicity, other elements from the plasmid, such as phleomycin and ampicillin resistance genes and the bacterial replication origin, were omitted. The drawing is not to scale. (B) PCR assay demonstrating integration of the full silencing cassette in transformants M9 and M11 transformed with pC6-RNAi. PCR products were subjected to electrophoresis in agarose gels. Lane WT: wild type strain *P*. *roqueforti* CECT 2905; lane E: *P*. *roqueforti* CECT 2905 containing empty pJL43-RNAi vector; lane S: Standard GeneRuler 1 kb DNA Ladder (Fermentas). Relevant sizes expressed in kb are shown at left. (C) qRT-PCR analysis of the expression of *pcz1* in *P*. *roqueforti* CECT 2905 (WT), *P*. *roqueforti* CECT 2905 containing empty pJL43-RNAi vector (E) and RNAi-silenced transformants M9 and M11. RNA was extracted from strains grown 5 days in YES medium. See Materials and Methods for other details. Error bars represent the standard deviation of three replicates in three different experiments. The differences were considered statistically significant at *P* < 0.05 (*) by using the Student’s *t*-test.

To functionally characterize the role of *pcz1*, RNA-mediated gene silencing was employed. For this purpose, *P*. *roqueforti* CECT 2905 was transformed with plasmid pC6-RNAi (see Materials and methods). After transformation, 46 phleomycin-resistant transformants were obtained. Fifteen of them were randomly selected and subjected to a preliminary screening by RT-PCR (data not shown). Compared with the wild-type strain, two of these transformants (named M9 and M11) showed dramatic reductions in the levels of *pcz1* transcript. To determine the amount of down-regulation in strains M9 and M11, qRT-PCR analyses were performed (Fig. 2). The transformants strains M9 and M11 showed approximately 39.4- and 31.4- fold decrease in *pcz1* transcripts compared with wild-type *P*. *roqueforti*. The presence of the full silencing cassette in these transformants was also confirmed (Fig. 2). Taken together, these data confirm the successful knockdown of *pcz1* in transformants M9 and M11. These transformants were used for further analysis.

### *pcz1* silenced transformants show abnormal phenotype

**Fig 3:**
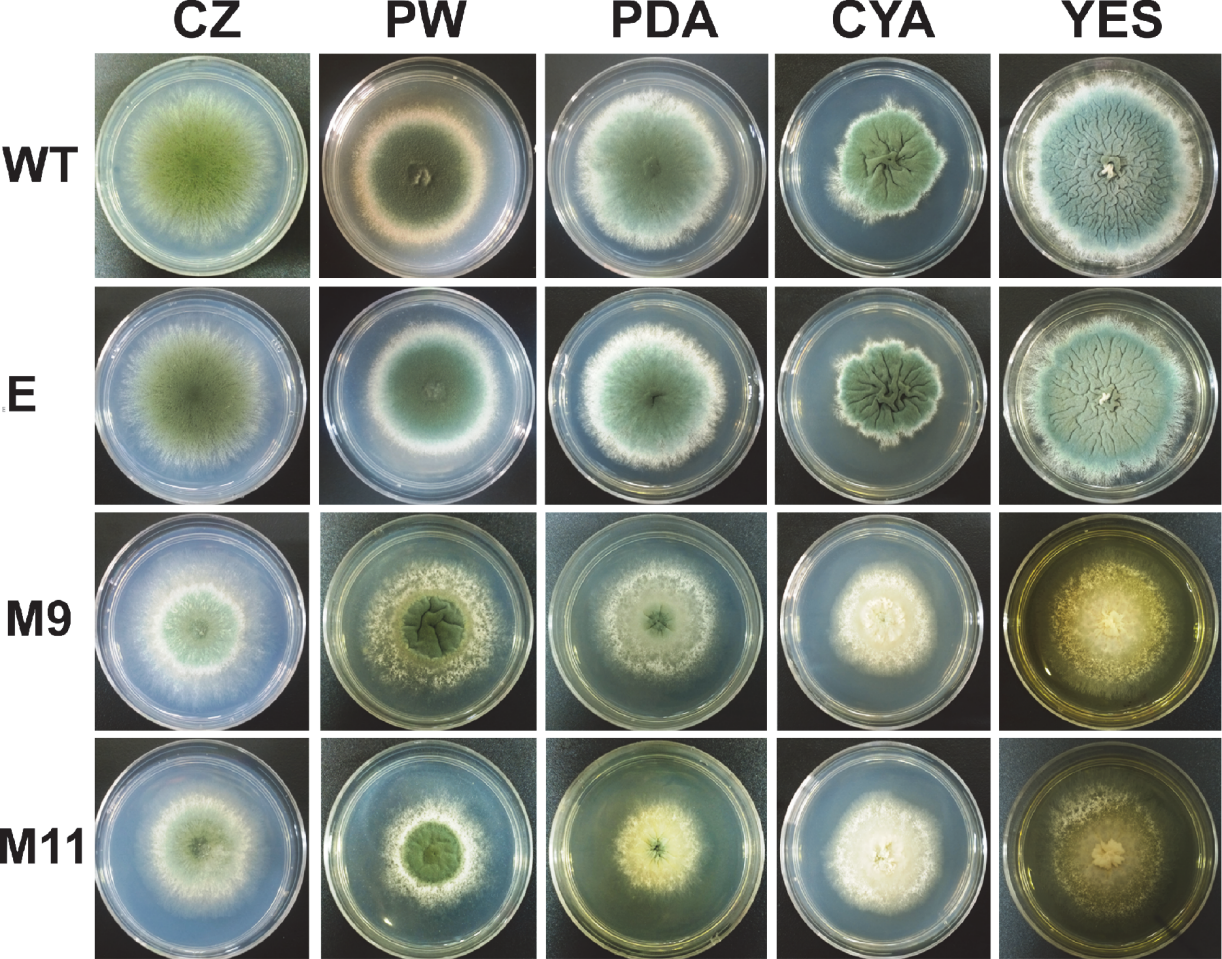
Phenotypes of different strains of *P*. *roqueforti*. The colonies were grown for 7 days at 28° C in Czapek (CZ), Power (PW), PDA, CYA, and YES media. WT: *P*. *roqueforti* CECT 2905; M9 and M11: *pcz1* silenced transformants; E: *P*. *roqueforti* CECT 2905 containing empty pJL43-RNAi vector. Note that the latter strain was undistinguishable from the wild-type strain.

The phenotype of transformants M9 and M11 was observed in several culture media (Fig. 3). Whereas wild-type *P*. *roqueforti* or the strain containing empty pJL43-RNAi vector showed the typical deep green color associated with normal sporulation, transformants M9 and M11 appeared to be whiter (Fig. 3). This effect can be clearly observed in PDA, CYA, YES and Czapek media (Fig. 3). In addition, the colonies of these transformants were smaller than the wild-type strain (Fig. 3). Therefore, the silencing of *pcz1* in the transformants altered the normal phenotype of *P*. *roqueforti*. It should be noted that on PW, PDA and YES media, the transformants M9 and M11 show slight differences in their phenotypes (Fig. 3). This could be due to different levels of decrease in *pcz1* transcripts in the transformants (see above).

### RNA-mediated silencing of *pcz1* decreased apical extension in *P*. *roqueforti*

**Fig 4:**
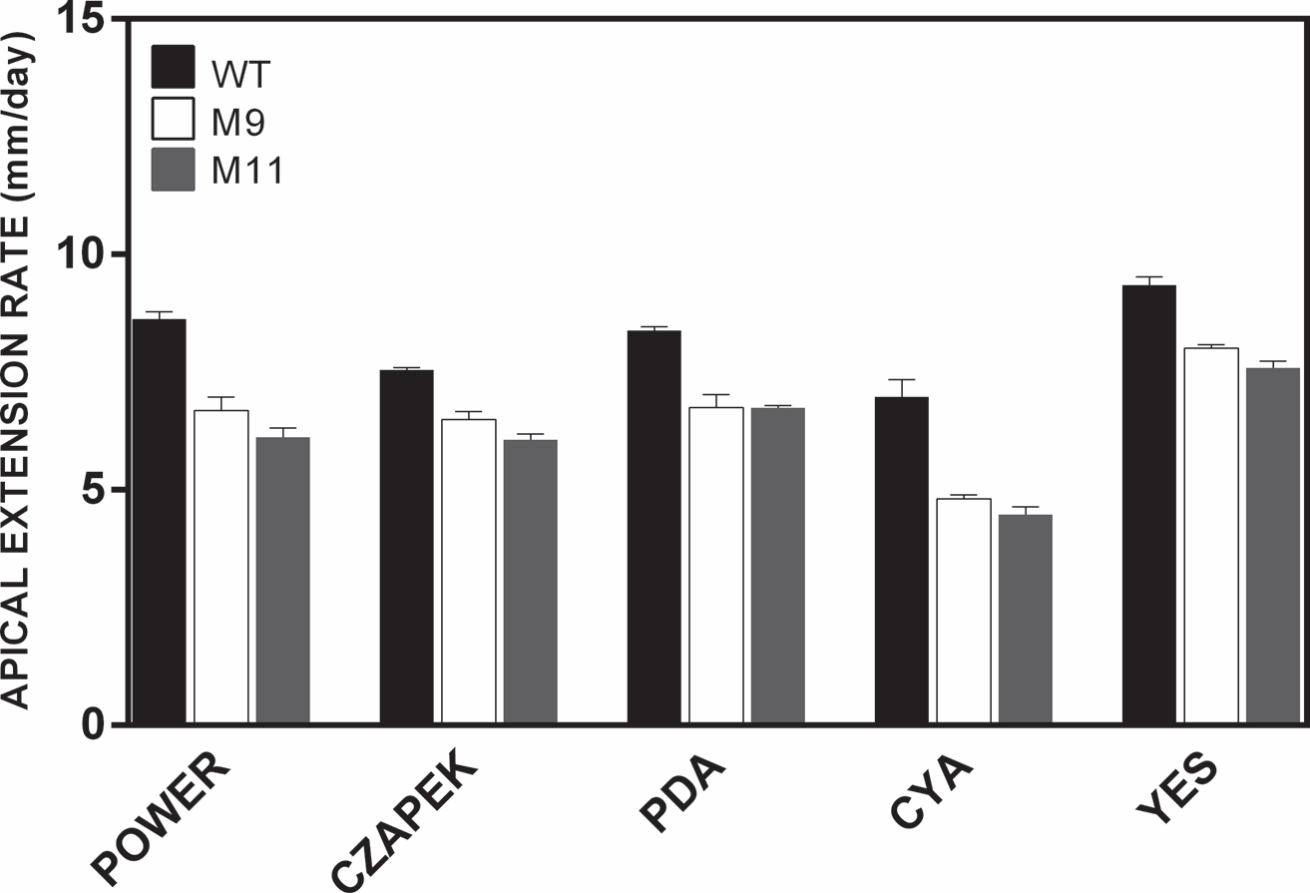
Apical extension rates (mm/day) of *P*. *roqueforti* CECT 2905 (WT) and transformants M9 and M11. Five different media (Power, Czapek, PDA, CYA and YES) were used. Error bars represent the standard deviation of three replicates in three different experiments. Apical extension rate of *P*. *roqueforti* containing empty pJL43-RNAi vector was statistically indistinguishable from the wild-type strain (not shown).

Transformants M9 and M11 showed a reduced rate of growth in all the media tested (Fig. 4). For example, the rate of growth of transformants M9 and M11 in CYA ranged between 4.56 and 4.80 mm/day, while the wild-type strain showed a rate of approximately 6.96 mm/day (Fig. 4). A similar behavior was observed in all other media tested. Expressed as a percentage and depending on the medium, the transformants grew at a rate of between 65.5 and 83% of the wild-type strain. These results suggest that *pcz1* plays a role in the vegetative growth of *P*. *roqueforti*, positively regulating this process.

### RNA-mediated silencing of *pcz1* strongly repressed conidiation in *P*. *roqueforti*

**Fig 5:**
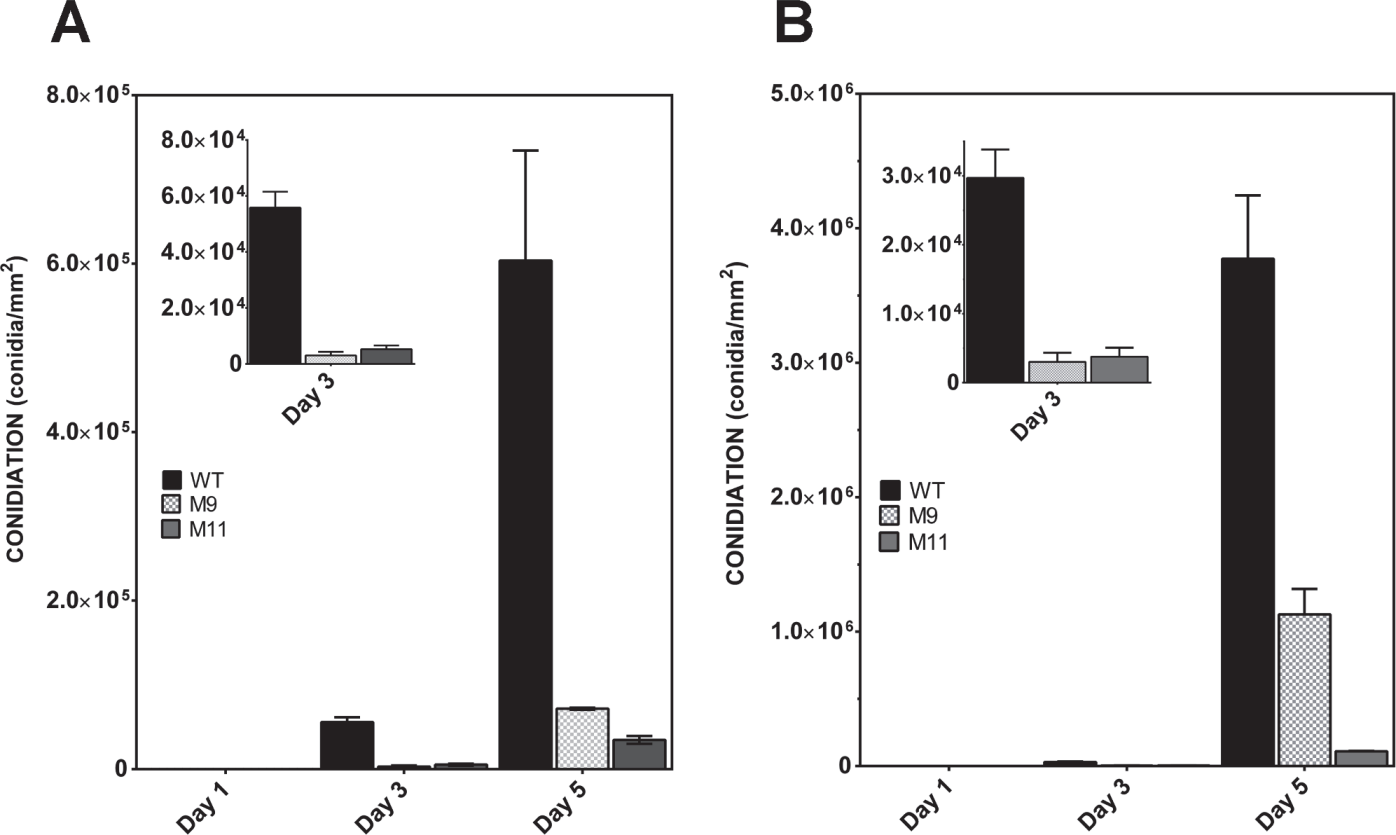
Conidial production of *P*. *roqueforti* CECT 2905 (WT) and transformants M9 and M11. Measurements were performed in two different media: Czapek minimal medium (A) and Power (B), a medium optimized for sporulation. Fungi were grown for 1, 3 and 5 days, and spores were collected as described in Materials and Methods. Error bars represent the standard deviation of three replicates in three independent experiments. For a clearer visualization, day 3 is also shown as an inset plot in A and B. Conidial production of *P*. *roqueforti* containing empty pJL43-RNAi vector was statistically indistinguishable from the wild-type strain (not shown).

The silencing of *pcz1* in *P*. *roqueforti* resulted in a drastic reduction in conidia formation (Fig. 5). For example, at 3 days of growth, transformants grown in Czapek (a minimal medium) produced between 5.4% and 9.4% of the number of conidia of the wild-type strain, whereas the same transformants grown on Power medium (a medium optimized for sporulation) produced up to 11.4% of the conidia produced by the wild-type strain. A similar behavior was observed at 5 days of growth. At this time, transformants grown on Czapek produced 5.8% and 11.7% of the number of conidia observed in the wild-type strain, whereas when grown on Power, they produced up to 19.1% of the conidia produced by the wild-type strain. These results indicate that the *pcz1* allele has a strong and positive role in conidiation.

### RNA-mediated silencing of *pcz1* decreased the expression of *brlA*, *abaA* and *wetA* in *P*. *roqueforti*

**Fig 6:**
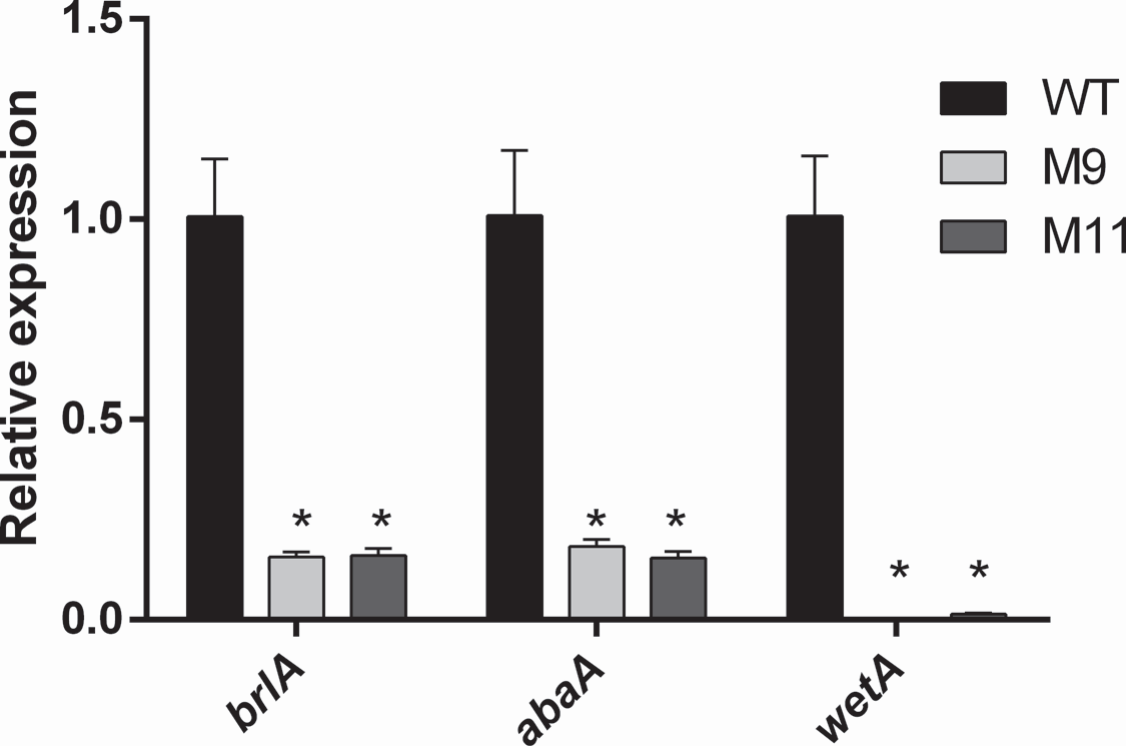
qRT-PCR analysis of the expression of *brlA*, *abaA* and *wetA* in *P*. *roqueforti* CECT 2905 (WT) and transformants M9 and M11. The strains were grown for 5 days at 28° C in Power medium. Total RNA extractions and qRT-PCR experiments were done as described in Materials and Methods. Error bars represent the standard deviation of three replicates in three different experiments. The differences were considered statistically significant at *P* < 0.05 (*) by using the Student’s *t*-test.

The current model of genetic control of conidiation (mainly based on studies performed in *Aspergillus* spp.) involves a network of regulators, which lead to a central conidiation pathway, composed of the genes *brlA*, *abaA* and *wetA* encoding transcription factors that control sporulation [23]. Because of the severe reduction of conidiation under *pcz1* silencing, we quantified the expression of these genes by qRT-PCR (Fig. 6). Compared with wild-type *P*. *roqueforti*, transformants M9 and M11 showed a drastic reduction of *brlA* transcripts (6.4- and 6.3-fold decrease, respectively), *abaA* transcripts (5.5- and 6.5-fold decrease, respectively), and *wetA* transcripts (304- and 67-fold decrease, respectively), suggesting that Pcz1 regulates positively the expression of conidiation-specific genes.

### RNA-mediated silencing of *pcz1* promotes conidial germination in *P*. *roqueforti*

**Fig 7:**
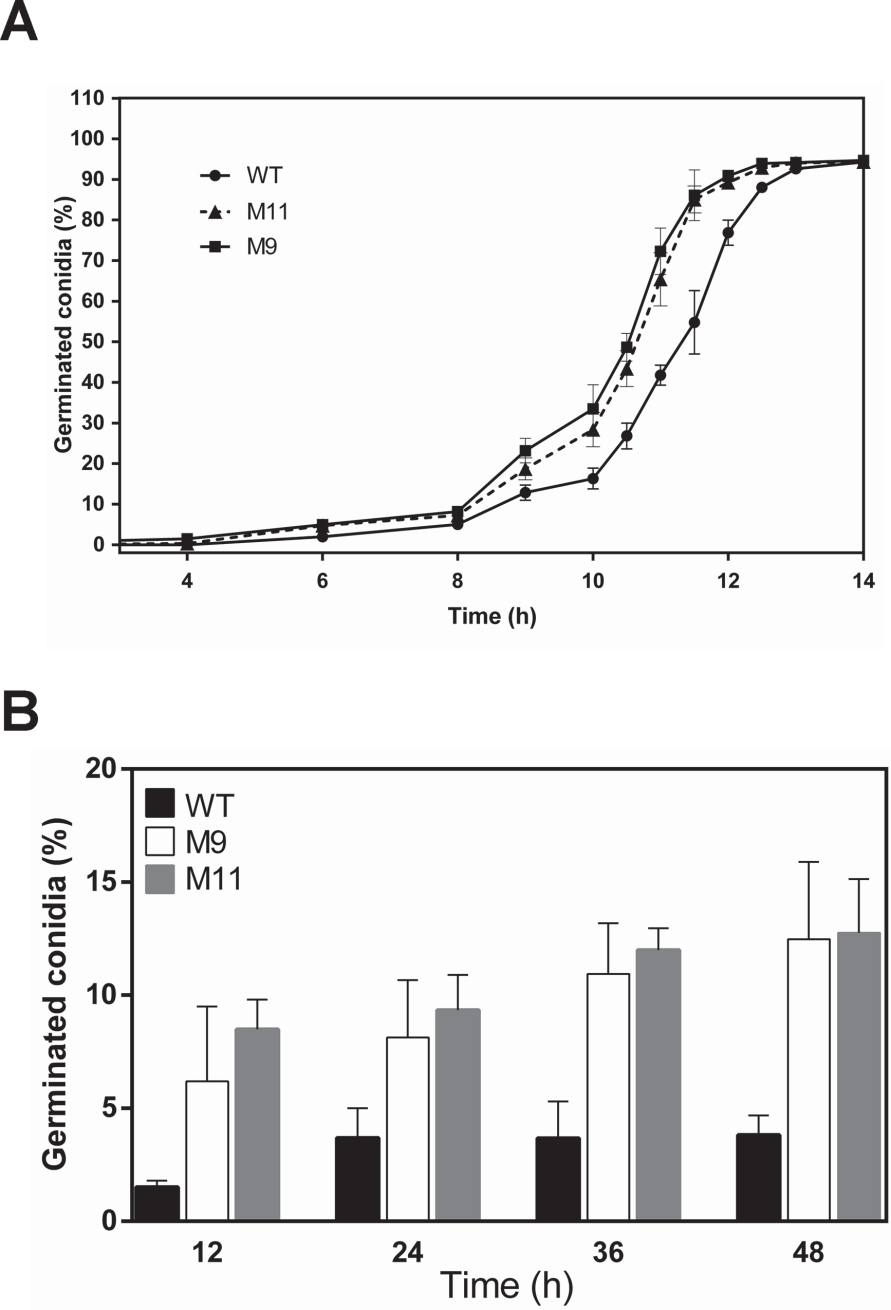
Germination rates of *P*. *roqueforti* CECT 2905 (WT) and transformants M9 and M11. (A) Germination kinetics in CM rich medium, represented as the percentage of germinated conidia vs. hours of incubation. Error bars represent the standard deviation of three replicates in three independent experiments. When the same experiment was performed in Czapek minimal medium, the same behavior was observed (not shown). (B) Germination in Czapek minimal medium lacking a carbon source. Percentage of germinated conidia after 12, 24, 36 and 48 hours of incubation is plotted. Error bars represent the standard deviation of three replicates in three independent experiments. Both in (A) and (B), no differences were observed between the wild-type strain and *P*. *roqueforti* containing empty pJL43-RNAi vector (not shown).

In addition to conidiation, *pcz1* also demonstrated a role in the control of conidial germination in *P*. *roqueforti*. Fig. 7 shows the germination kinetics of the wild-type strain and the *pcz1*-silenced transformants. As expected, all strains followed a sigmoidal pattern of conidiation. However, in the silenced transformants, the germination process was earlier compared with the wild-type strain. Thus at 10 hours, whereas approximately 16.3% of wild-type conidia had germinated, 33.5 and 28.4% of conidia from strains M9 and M11 had germinated, respectively. These differences were maintained throughout the exponential phase of germination, and at 12 hours, whereas conidial germination from strains M9 and M11 reached approximately 93.1%, the percentage of conidial germination of the wild-type strain was approximately 76.9%.

### RNA-mediated silencing of *pcz1* triggers conidial germination in the absence of a carbon source

Taking into account the differences observed in germination kinetics, and because conidial germination is triggered mainly by the presence of a carbon source [24,25], we tested whether the strains with attenuated *pcz1* expression were able to germinate in a medium lacking a carbon source. Consistently, and for all the times assayed (12 to 48 hours), transformants M9 and M11 showed enhanced conidial germination compared with the wild-type strain. Approximately 12.7% of conidia from transformants were able to germinate after 48 h in minimal Czapek medium lacking a carbon source, whereas at the same time, 3.8% of conidia from the *P*. *roqueforti* wild type strain had germinated (Fig. 7). These results indicate that the attenuation of *pcz1* allows germination in the absence of carbon source, suggesting that *pcz1* is a negative regulator of conidial germination, probably intervening in a pathway that senses carbon sources.

## Discussion

*Penicillium roqueforti* is used throughout the world in the production of ripened blue-veined cheeses, but unlike model fungal organisms (i.e., *Aspergillus* spp.) or other industrial filamentous fungi (i.e., *P*. *chrysogenum*, currently known as *P*. *rubens*), the functional characterization of genes from *P*. *roqueforti* using genetics strategies is still in its infancy. Thus, this work is one of the few examples of functional characterization of a gene in this fungus.

**Fig 8:**
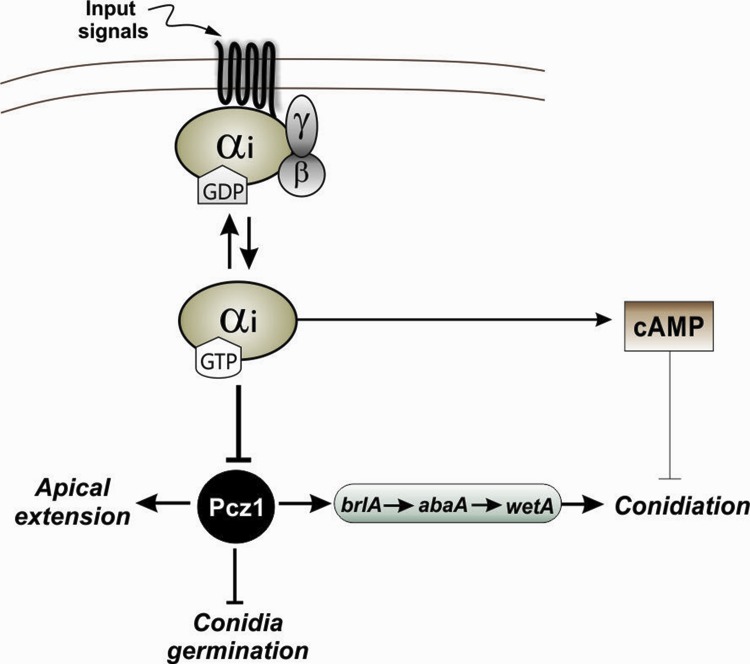
A working model showing developmental processes controlled by *pcz1* in *P*. *roqueforti*. The model includes the suggested relationship between *pcz1* and the Gαi subunit, and the existence of cAMP-independent and cAMP-dependent pathways related to conidiation. The thickness of the line indicates the magnitude of the effect. The model includes effects inferred by experimental data from this work and previous results [8,9]. See the main text for details.

According to the data described in this work, RNA-mediated silencing of *pcz1* strongly represses conidiation (Fig. 5) and decreases the expression of *brlA*, *abaA* and *wetA* (Fig. 6). Activation of brlA is a key step of conidiation. BrlA is a transcription factor that during the middle stage of conidiation activates abaA, an essential component for differentiation and function of phialides [23]. Also, BrlA activates *wetA* during the late phase of conidiation; *wetA* plays an important role for the synthesis of spore cell wall [23]. Accordingly, our results suggest that *pcz1* stimulates sporulation through the positive regulation of conidiation-specific genes expression (Fig. 8). Further work is necessary to determine if this is the result of a direct transcriptional regulation of Pcz1 or of an indirect effect.

Upstream of the conidiation central pathway, there are a group of activators of BrlA (*flbB*-*flbE*), which are in turn negatively regulated by the repressor protein SfgA [5,23]. Interestingly, SfgA is a C6 protein, but conversely to Pcz1, SfgA is a repressor of conidiation [5]. In *A*. *nidulans*, overexpression of *sfgA* resulted in reduced levels of mRNA of *brlA*, suggesting that SfgA inhibits the expression of this gene [5]. Therefore, the opposite roles of SfgA and Pcz1 in conidiation can be explained by their opposite roles in the regulation of *brlA* expression. In addition to *sfgA*, three other C6 genes (*oefC* from *A*. *nidulans* and MoCOD1 and MoCOD2 from Magnaporthe oryzae) are involved in the control of conidiation [26,27]. However, their exact roles in the process have not yet been established.

In *Aspergillus*, *sfgA* has also been proposed as a regulator of vegetative growth, and to date, it is the only C6 gene involved in the control of this process. SfgA negatively regulates FlbA, a regulator of G-protein signaling (RGS) that stimulates the intrinsic GTPase activity of a Gαi subunit. Thus, SfgA helps in the promotion of vegetative growth through Gαi signaling in *Aspergillus* [23,25]. Interestingly, the attenuation of *pcz1* represses vegetative growth in *P*. *roqueforti* (Fig. 4), suggesting that Pcz1 is a positive regulator of this process. This behavior of *pcz1* agrees with the previous description of the role of G-protein signaling (particularly the Gαi subunit) in *P*. *roqueforti* and other fungi, such as *P*. *chrysogenum*, *Fusarium sporotrichioides* and *F*. *fujikuroi* [8,28,29]. In these organisms, and contrary to what has been described in *Aspergillus*, Gαi signaling has a negative effect on the apical growth rate. Because the Gαi subunit downregulates *pcz1* in *P*. *roqueforti* (Fig. 1), we suggest a model relating both regulators and their effects on vegetative growth in *P*. *roqueforti* (Fig. 8). Regarding the model, we observed that depending on the culture medium used, the attenuation of the expression of *pcz1* results in approximately 65–83% apical extension rate compared with wild-type *P*. *roqueforti* (Fig. 4). The effect produced by the transformation of *P*. *roqueforti* with the dominant active Gαi protein was previously estimated to be approximately 40–70% of the apical extension rate of the wild-type strain [8]. Therefore, *pcz1* may account for all or most of the control of apical extension rate produced by the dominant active Gαi protein (Fig. 8).

To the best of our knowledge, only one C6 gene has been functionally related to conidial germination in fungi to date. In M. oryzae, the absence of the MoCOD1 gene severely reduced the germination rate of its conidia [27], indicating that this C6 gene may be a positive regulator of conidial germination. In our case, we observed that the attenuation of *pcz1* increased the germination rate of conidia in *P*. *roqueforti* (Fig. 7), suggesting that contrary to MoCOD1, *pcz1* may be a negative regulator of conidial germination. Importantly, this effect is maintained even in the absence of a carbon source (Fig. 7). Initiation and completion of germination require the sensing of external signals, particularly a carbon source, which is usually the signal for conidial germination [25]. The presence of organic molecules is sensed by a suitable receptor and transmitted through the Gα-cAMP signaling pathway to protein kinase A (PkaA), which phosphorylates target proteins, thus beginning the conidial germination process [25]. Interestingly in a previous report, it was demonstrated that the Gαi protein has a positive effect on conidial germination in the absence of a carbon source in *P*. *roqueforti* [10], and now we have shown that Gαi downregulates *pcz1* (Fig. 1). Taken together, these results suggest a model where *pcz1* may be participating in the Gαi-signaling pathway that senses carbon sources and triggers conidial germination (Fig. 8). Further work is necessary to validate this suggestion.

Using the data obtained in this work and previously published data [8,9] we propose an integrated working model of the role of Pcz1 in developmental processes in *P*. *roqueforti* (Fig. 8), whose roles in vegetative growth and conidial germination were discussed above. Another interesting aspect of this model is related to conidiation. In *P*. *roqueforti*, Gαi controls conidiation through two pathways: cAMP-dependent and cAMP-independent pathways [9]. Specifically in this fungus, it has been demonstrated that an artificial increase in intracellular cAMP levels had a minor effect on conidiation (14–20% reduction of conidiation) compared with the effect produced by transformation with the dominant active Gαi protein (>99% reduction of conidiation; [9]). Therefore, in *P*. *roqueforti*, the repression of conidiation by Gαi is mainly (approximately 80%) through the cAMP-independent pathway [9]. Interestingly, and depending on the transformant and medium used, the attenuation of *pcz1* resulted in 80–94% less conidiation than in the parental strain (Fig. 5), which matches well with the magnitude of the expected impact of the cAMP-independent pathway. Therefore, and although further confirmatory experiments are necessary, it seems very probable that *pcz1* may be part of the cAMP-independent pathway that controls conidiation (Fig. 8). The same dual control of conidiation by Gαi through cAMP-dependent and cAMP-independent pathways has been observed in *A*. *nidulans* and *P*. *chrysogenum* [9,30]. In this context, it will be interesting to analyze the role of the orthologues of *pcz1* in these fungi in the future.

## Supporting Information

S1 Fig Dot blot assay showing the differential expression of *pcz1* in a subtractive library. The dot blot assay was carried out as described in Klagges et al. [20]. Briefly, the same nylon membrane containing purified plasmids from selected clones was hybridized with subtracted cDNA from wild-type *P*. *roqueforti* (A) or subtracted cDNA from *P*. *roqueforti* pga7 (B). Those clones harboring putative differentially expressed cDNAs in the wild-type strain should hybridize only to A. Numbers in red are nomenclature of the clones. Clone 321 was differentially expressed in the wild-type strain (highlighted in the red box) and contains *pcz1* cDNA. C+: Positive control for hybridization.
(TIF)

S2 Fig Multiple alignment of Pcz1 and its orthologues from 81 fungal species from the phylum Ascomycota. Alignment was performed with Clustal Omega using default parameters. Full sequences were aligned, but only the region spanning the Zn(II)_2_Cys_6_ DNA binding domain is shown. Asterisks indicate fully conserved residues. The six key conserved cysteines are shown in blue. Sequence belonging to Pcz1 is bolded and underlined. At the left, the name of each organism and the Genbank accession number for each sequence is indicated. The names of some fungi are abbreviated: *M*. *anisopliae*: *Metarhizium anisopliae*; *S*. *chlorohalonata*: *Stachybotrys chlorohalonata*; *P*. *destructans*: *Pseudogymnoascus destructans*; *S*. *sclerotiorum*: *Sclerotinia sclerotiorum*; *M*. *thermophila*: *Myceliophthora thermophila*; *L*. *maculans*: *Leptosphaeria maculans*; *C*. *apollinis*: *Coniosporium apollinis*; *B*. *compniacensis*: *Baudoinia compniacensis*; *C*. *psammophila*: *Cladophialophora psammophila*; *T*. *stipitatus*: *Talaromyces stipitatus*; *P*. *brasiliensis*: *Paracoccidioides brasiliensis*; *C*. *posadasii*: *Coccidioides posadasii*; *T*. *interdigitale*: *Trichophyton interdigitale*; *T*. *verrucosum*: *Trichophyton verrucosum*; *C*. *yegresii*: *Cladophialophora yegresii*; *C*. *carrionii*: *Cladophialophora carrionii*; *P*. *fijiensis*: *Pseudocercospora fijiensis*.
(DOCX)

S1 Table Primers used in qRT-PCR experiments. (DOCX)

S2 Table Correlation coefficient (R^2^), slope and efficiency of calibration curves obtained for the genes analyzed by qRT-PCR. (DOCX)
