# Supplementary figures and images for: The pcz1 Gene, which Encodes a Zn(II)2Cys6 Protein, Is Involved in the Control of Growth, Conidiation, and Conidial Germination in the Filamentous Fungus Penicillium roqueforti
Source: PLoS One. 2015 Mar 26;10(3):e0120740. doi: 10.1371/journal.pone.0120740 (PMC4374774; doi:10.1371/journal.pone.0120740)

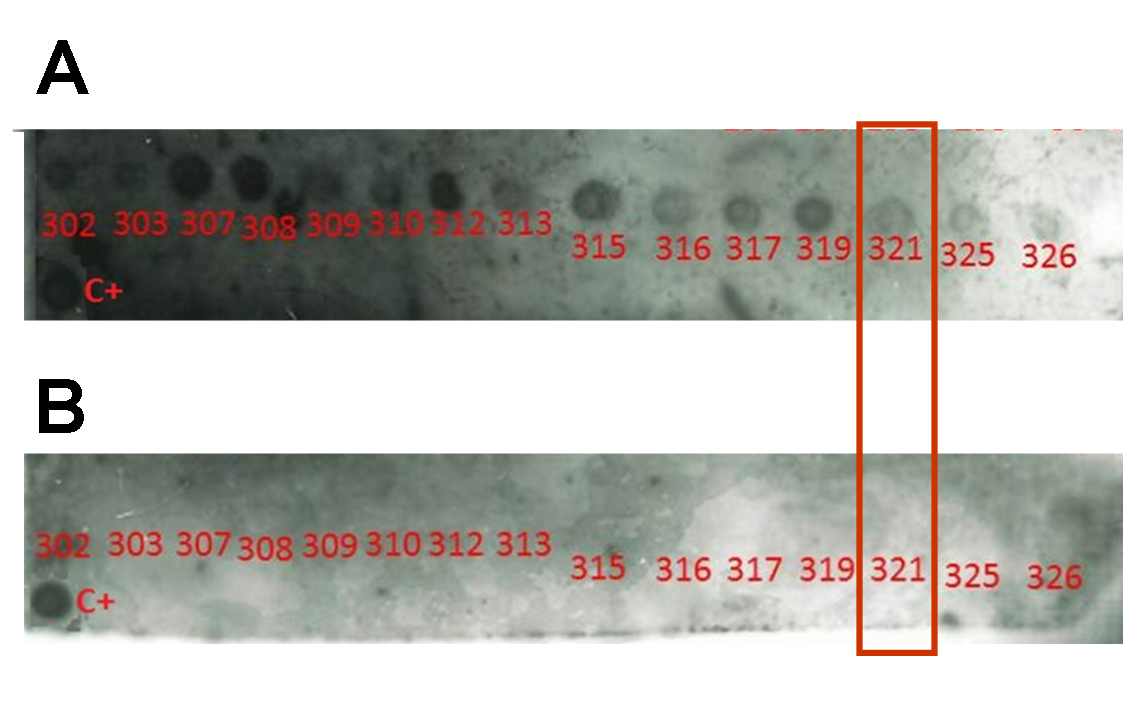

Supplement: S1 Fig — The dot blot assay was carried out as described in Klagges et al. [20]. Briefly, the same nylon membrane containing purified plasmids from selected clones was hybridized with subtracted cDNA from wild-type P. roqueforti (A) or subtracted cDNA from P. roqueforti pga7 (B). Those clones harboring putative differentially expressed cDNAs in the wild-type strain should hybridize only to A. Numbers in red are nomenclature of the clones. Clone 321 was differentially expressed in the wild-type strain (highlighted in the red box) and contains pcz1 cDNA. C+: Positive control for hybridization. (TIF) [file pone.0120740.s001.tif]
