# Supplementary material for: The pcz1 Gene, which Encodes a Zn(II)2Cys6 Protein, Is Involved in the Control of Growth, Conidiation, and Conidial Germination in the Filamentous Fungus Penicillium roqueforti
Source: PLoS One. 2015 Mar 26;10(3):e0120740. doi: 10.1371/journal.pone.0120740 (PMC4374774; doi:10.1371/journal.pone.0120740)
Supplement: S2 Fig — Alignment was performed with Clustal Omega using default parameters. Full sequences were aligned, but only the region spanning the Zn(II)2Cys6 DNA binding domain is shown. Asterisks indicate fully conserved residues. The six key conserved cysteines are shown in blue. Sequence belonging to Pcz1 is bolded and underlined. At the left, the name of each organism and the Genbank accession number for each sequence is indicated. The names of some fungi are abbreviated: M. anisopliae: Metarhizium anisopliae; S. chlorohalonata: Stachybotrys chlorohalonata; P. destructans: Pseudogymnoascus destructans; S. sclerotiorum: Sclerotinia sclerotiorum; M. thermophila: Myceliophthora thermophila; L. maculans: Leptosphaeria maculans; C. apollinis: Coniosporium apollinis; B. compniacensis: Baudoinia compniacensis; C. psammophila: Cladophialophora psammophila; T. stipitatus: Talaromyces stipitatus; P. brasiliensis: Paracoccidioides brasiliensis; C. posadasii: Coccidioides posadasii; T. interdigitale: Trichophyton interdigitale; T. verrucosum: Trichophyton verrucosum; C. yegresii: Cladophialophora yegresii; C. carrionii: Cladophialophora carrionii; P. fijiensis: Pseudocercospora fijiensis. (DOCX) [file pone.0120740.s002.docx]

*Villosiclava virens* KDB11906 EIRKLRACLRCKFLKKTCDKGEPCAGCQPSHARLWQVPCTRIDIKD

*Metarhizium acridum* XP_007808983 EIRKLRACLRCKFLKKTCDKGEPCAGCQPSHARLWQVPCTRIDIKD

*Metarhizium robertsii* EXV05783 EIRKLRACLRCKFLKKTCDKGEPCAGCQPSHARLWQVPCTRIDIKD

*M. anisopliae* XP_007818348 EIRKLRACLRCKFLKKTCDKGEPCAGCQPSHARLWQVPCTRIDIKD

*S. chlorohalonata* KFA61496 EIRKLRACLRCKFLKKTCDKGEPCAGCQPSHARLWQVPCTRIDIKD

*Stachybotrys chartarum* KEY66824 EIRKLRACLRCKFLKKTCDKGEPCAGCQPSHARLWQVPCTRIDIKD

*Ophiocordyceps sinensis* EQL01177 EIRKLRACLRCKFLKKTCDKGEPCAGCQPSHARLWQVPCTRIDIKD

*Nectria haematococca* XP_003052574 EIRKLRACLRCKFLKKTCDKGEPCAGCQPSHARLWQVPCTRIDIKD

*Fusarium oxysporum* EXM09000 EIRKLRACLRCKFLKKTCDKGEPCAGCQPSHARLWQVPCTRIDIKD

*P. destructans* ELR08710 EIRKLRACLRCKFLKKTCDKGEPCAGCQPSHARLWQVPCTRIDIKD

*Glarea lozoyensis* XP_008079137 EIRKLRACLRCKFLKKTCDKGEPCTGCQPSHARLWQVPCTRIDIKD

*Sclerotinia boreales* ESZ99356 EIRKLRACLRCKFLKKTCDKGEPCAGCQPSHARLWQVPCTRIDIKD

*Botrytis cinerea* EMR84819 EIRKLRACLRCKFLKKTCDKGEPCAGCQPSHARLWQVPCTRIDIKD

*S. sclerotiorum* XP_001598084 EIRKLRACLRCKFLKKTCDKGEPCAGCQPSHARLWQVPCTRIDIKD

*Pestalotiopsis fici* XP_007840956 EIRKLRACLRCKFLKKTCDTGEPCNGCQPSHARLWQVPCTRIDIKD

*Neurospora crassa* XP_958804 EIRKLRACLRCKFLKKTCDKGEPCAGCQPSHARLWQVPCTRIDIKD

*Neurospora tetrasperma* EGO54670 EIRKLRACLRCKFLKKTCDKGEPCAGCQPSHARLWQVPCTRIDIKD

*Sordaria macrospora* XP_003350609 EIRKLRACLRCKFLKKTCDKGEPCAGCQPSHARLWQVPCTRIDIKD

*Thielavia terrestres* XP_003650468 EIRKLRACLRCKFLKKTCDKGEPCAGCQPSHARLWQVPCTRIDIKD

*M. thermophila* XP_003664132 EIRKLRACLRCKFLKKTCDKGEPCAGCQPSHARLWQVPCTRIDIKD

*Chaetomium globosum* XP_001227037 EIRKLRACLRCKFLKKTCDKGEPCAGCQPSHARLWQVPCTRIDIKD

*Podospora anserina* XP_001906056 EIRKLRACLRCKFLKKTCDKGEPCAGCQPSHARLWQVPCTRIDIKD

*Togninia minima* XP_007915414 EIRKLRACLRCKFLKKTCDKGEPCAGCQPSHARLWQVPCTRIDIKD

*Phaeosphaeria nodorum* XP_001797030 EIRKLRACLRCKFLKKTCDKGDPCAGCQPSHARLWQVPCTRIDIKD

*L. maculans* XP_003843817 EIRKLRACLRCKFLKKTCDKGDPCAGCQPSHARLWQVPCTRIDIKD

*Pyrenophora teres* XP_003296191 EIRKLRACLRCKFLKKTCDKGDPCAGCQPSHARLWQVPCTRIDIKD

*P. tritici-repentis* XP_001931826 EIRKLRACLRCKFLKKTCDKGDPCAGCQPSHARLWQVPCTRIDIKD

*Setosphaeria turcica* XP_008020387 EIRKLRACLRCKFLKKTCDKGDPCAGCQPSHARLWQVPCTRIDIKD

*Bipolaris sorokiniana* XP_007695810 EIRKLRACLRCKFLKKTCDKGDPCAGCQPSHARLWQVPCTRIDIKD

*Bipolaris zeicola* XP_007714488.1 EIRKLRACLRCKFLKKTCDKGDPCAGCQPSHARLWQVPCTRIDIKD

*Bipolaris victoriae* EUN28037 EIRKLRACLRCKFLKKTCDKGDPCAGCQPSHARLWQVPCTRIDIKD

*Bipolaris maydis* EMD93448 EIRKLRACLRCKFLKKTCDKGDPCAGCQPSHARLWQVPCTRIDIKD

*Bipolaris oryzae* XP_007691700 EIRKLRACLRCKFLKKTCDKGDPCAGCQPSHARLWQVPCTRIDIKD

*C. apollinis* XP_007782137 EIRKLRACLRCKFLKKTCDKGDPCAGCQPSHARLWQVPCTRVDIKD

*Aureobasidium pullulans* KEQ69030 EIRKLRACLRCKFLKKTCDKGNPCGGCRPSHARLWQVPCTRIDIKD

*Aureobasidium melanogenum* KEQ59830 EIRKLRACLRCKFLKKTCDKGNPCGGCRPSHARLWQVPCTRIDIKD

*Aureobasidium subglaciale* KER00645 EIRKLRACLRCKFLKKTCDKGNPCGGCRPSHARLWQVPCTRIDIKD

*Macrophomina phaseolina* EKG13256 EIRKLRACLRCKFLKKTCDKGDPCAGCQPSHARLWQVPCTRIDIKD

*Neofusicoccum parvum* XP_007581338 EIRKLRACLRCKFLKKTCDKGDPCAGCQPSHARLWQVPCTRIDIKD

*B. compniacensis* XP_007674584 EIRKLRACLRCKFLKKTCDKGEPCGGCRPSHARLWQVPCTRMDIKD

*Zymoseptoria tritici* XP_003853786 EIRKLRACLRCKFLKKTCDKGEPCGGCRPSHARLWMVPCTRMDIKD

*P fijiensis* XP_007924439 EIRKLRACLRCKFLKKTCDKGEPCGGCRPSHARLWQVPCTRMDIKD

*Sphaerulina musiva* EMF13509 EIRKLRACLRCKFLKKTCDKGEPCGGCRPSHARLWQVPCTRMDIKD

*Endocarpon pusillum* XP_007805938 EIRKLRACLRCKFLKKTCDKGEPCAGCKPSHARLWQVPCTRIDIKE

*Cyphellophora europaea* ETN46056 EIRKLRACLRCKFLKKTCDKGEPCAGCRPSHARLWQVPCTRIDIKE

*Exophiala aguamarina* KEF53709 EIRKLRACLRCKFLKKTCDKGEPCAGCQPSHARLWQVPCTRIDIKE

*C. psammophila* XP_007741977 EIRKLRACLRCKFLKKTCDKGEPCAGCQPSHARLWQVPCTRIDIKE

*C. carrionii* ETI25064 EIRKLRACLRCKFLKKTCDKGEPCAGCQPSHARLWQVPCTRIDIKE

*C. yegresii* XP_007756461 EIRKLRACLRCKFLKKTCDKGEPCAGCQPSHARLWQVPCTRIDIKE

*Capronia epimyces* XP_007731399 EIRKLRACLRCKFLKKTCDKGEPCAGCQPSHARLWQVPCTRIDIKE

*Exophiala dermatitidis* EHY58693 EIRKLRACLRCKFLKKTCDKGEPCAGCQPSHARLWQVPCTRIDIKE

*Arthroderma otae* XP_002844501 EIRKLRACLRCKFLKKTCDKGEPCTGCQPSHARLWQVPCTRIDIKE

*Microsporum gypseum* XP_003170929 EIRKLRACLRCKFLKKTCDKGEPCTGCQPSHARLWQVPCTRIDIKE

*Arthroderma benhamiae* XP_003010028 EIRKLRACLRCKFLKKTCDKGEPCTGCQPSHARLWQVPCTRIDIKE

*T. verrucosum* XP_003019726 EIRKLRACLRCKFLKKTCDKGEPCTGCQPSHARLWQVPCTRIDIKE

*Trichophyton rubrum* EZG09816.1 EIRKLRACLRCKFLKKTCDKGEPCTGCQPSHARLWQVPCTRIDIKE

*Trichophyton soudanense* EZF77168 EIRKLRACLRCKFLKKTCDKGEPCTGCQPSHARLWQVPCTRIDIKE

*T. interdigitale* EZF31873 EIRKLRACLRCKFLKKTCDKGEPCTGCQPSHARLWQVPCTRIDIKE

*Trichophyton tonsurans* EGD97993 EIRKLRACLRCKFLKKTCDKGEPCTGCQPSHARLWQVPCTRIDIKE

*Trichophyton equinum* EGE04993 EIRKLRACLRCKFLKKTCDKGEPCTGCQPSHARLWQVPCTRIDIKE

*C. posadasii* XP_003066450 EIRKLRACLRCKFLKKTCDKGEPCAGCKPSHARLWQVPCTRIDIKE

*Coccidioides immitis* XP_001246795 EIRKLRACLRCKFLKKTCDKGEPCAGCKPSHARLWQVPCTRIDIKE

*Uncinocarpus reesii* XP_002541074 EIRKLRACLRCKFLKKTCDKGEPCAGCKPSHARLWQVPCTRIDIKE

*Ajellomyces capsulatus* EEH07230 EIRKLRACLRCKFLKKTCDKGEPCAGCQPSHARLWQVPCTRIDIKE

*P. brasiliensis* EEH20849 EIRKLRACLRCKFLKKTCDKGEPCAGCQPSHARLWQVPCTRIDIKE

*Talaromyces marneffei* XP_002151174 EIRKLRACLRCKFLKKTCDKGEPCAGCQPSHARLWQVPCTRIDIKE

*T. stipitatus* XP_002341865 EIRKLRACLRCKFLKKTCDKGEPCAGCQPSHARLWQVPCTRIDIKE

*Penicillium oxalicum* EPS29014 EIRKLRACLRCKFLKKTCDKGEPCAGCQPSHARLWQVPCTRIDIKE

*Penicillium digitatum* EKV13150 EIRKLRACLRCKFLKKTCDKGEPCAGCQPSHARLWQVPCTRIDIKE

***Penicillium roqueforti* CDM35469 EIRKLRACLRCKFLKKTCDKGEPCAGCQPSHARLWQVPCTRIDIKE**

*Penicillium rubens* XP_002565184 EIRKLRACLRCKFLKKTCDKGEPCAGCQPSHARLWQVPCTRIDIKE

*Aspergillus ruber* EYE99876 EIRKLRACLRCKFLKKTCDKGEPCAGCQPSHARLWQVPCTRIDIKE

*Byssochlamys spectabilis* GAD99062 EIRKLRACLRCKFLKKTCDKGEPCAGCQPSHARLWQVPCTRIDIKE

*Aspergillus nidulans* CBF85988 EIRKLRACLRCKFLKKTCDKGEPCAGCQPSHARLWQVPCTRIDIKE

*Aspergillus terreus* XP_001210781 EIRKLRACLRCKFLKKTCDKGEPCAGCQPSHARLWQVPCTRIDIKE

*Aspergillus oryzae* KDE79146 EIRKLRACLRCKFLKKTCDKGEPCAGCQPSHARLWQVPCTRIDIKE

*Aspergillus flavus* XP_002374144 EIRKLRACLRCKFLKKTCDKGEPCAGCQPSHARLWQVPCTRIDIKE

*Aspergillus kawachii* GAA87411 EIRKLRACLRCKFLKKTCDKGEPCAGCQPSHARLWQVPCTRIDIKE

*Aspergillus niger* EHA20824 EIRKLRACLRCKFLKKTCDKGEPCAGCQPSHARLWQVPCTRIDIKE

*Aspergillus clavatus* XP_001271908 EIRKLRACLRCKFLKKTCDKGEPCAGCQPSHARLWQVPCTRIDIKE

*Aspergillus fumigatus* KEY75841 EIRKLRACLRCKFLKKTCDKGEPCAGCQPSHARLWQVPCTRIDIKE

*Neosartorya fischeri* XP_001266982 EIRKLRACLRCKFLKKTCDKGEPCAGCQPSHARLWQVPCTRIDIKE

*******************.*:** **:******* *****:***:
