# Supplementary material for: The pcz1 Gene, which Encodes a Zn(II)2Cys6 Protein, Is Involved in the Control of Growth, Conidiation, and Conidial Germination in the Filamentous Fungus Penicillium roqueforti
Source: PLoS One. 2015 Mar 26;10(3):e0120740. doi: 10.1371/journal.pone.0120740 (PMC4374774; doi:10.1371/journal.pone.0120740)
Supplement: S1 Table — (DOCX) [file pone.0120740.s003.docx]

**S1 Table. Primers used in qRT-PCR experiments**

| Target gene | Name of the primer | Sequence (5`-3´) | Amplicon size (bp) |
| --- | --- | --- | --- |
| *pcz1* | qRT-pcz1-fw | GATTACGAACGCCACATCAC | 87 |
|  | qRT-pcz1-rv | GTGGGTGATGAACAGAGTGC |  |
| *brlA* | qRT-brlA-fw | GATCTCACCTCGAGCGAAAC | 81 |
|  | qRT-brlA-rv | ATAGTCTGGGAGGGGCATCT |  |
| *abaA* | qRT-abaA-fw | ATCTGCAGGTCCTCGACTCT | 77 |
|  | qRT-abaA-rv | TCGTTCTAATGCTGGCTCAC |  |
| *wetA* | qRT-wetA-fw | GAGCCTTGGTCTTGGAACTG | 90 |
|  | qRT-wetA-rv | TCAGCGTATCGTTCGTCAAC |  |
| β-tubulin | qRT-btub-fw | TCCAAGGTTTCCAGATCACC | 89 |
|  | qRT-btub-rv | GAACTCCTCACGGATCTTGG |  |
