# Supplementary material for: The pcz1 Gene, which Encodes a Zn(II)2Cys6 Protein, Is Involved in the Control of Growth, Conidiation, and Conidial Germination in the Filamentous Fungus Penicillium roqueforti
Source: PLoS One. 2015 Mar 26;10(3):e0120740. doi: 10.1371/journal.pone.0120740 (PMC4374774; doi:10.1371/journal.pone.0120740)
Supplement: S2 Table — (DOCX) [file pone.0120740.s004.docx]

**S2 Table. Correlation coefficient (R^2^), slope and efficiency of calibration curves obtained for the genes analyzed by qRT-PCR**

|  | β-tubulin | *pcz*1 | *brlA* | *abaA* | *wetA* |
| --- | --- | --- | --- | --- | --- |
| R^2^ | 0.980 | 0.982 | 0.990 | 0.992 | 0.990 |
| Slope | -3.331 | -3.179 | -3.233 | -3.502 | -3.508 |
| Efficiency (%) | 99.635 | 106.35 | 103.85 | 92.979 | 92.791 |
